# The transverse cervical artery cervical cutaneous branch flap: An anatomy-based nomenclature

**DOI:** 10.3389/fsurg.2022.1029065

**Published:** 2023-01-06

**Authors:** Chen Dong, Zhou Yu, Xianjie Ma

**Affiliations:** Department of Plastic Surgery, Xijing Hospital, Fourth Military Medical University, Xi’an, China

**Keywords:** transverse cervical artery, cutaneous branch, flap, anatomy, nomenclature

## Introduction

The transverse cervical artery (transverse artery of the neck or transverse colli artery) is an artery in the neck and a branch of the thyrocervical trunk, running at a higher level than the suprascapular artery (1). It passes transversely above the inferior belly of the omohyoid muscle to the anterior margin of the trapezius, beneath which it divides into superficial and deep branches (2). The branches from the transverse cervical artery are widely distributed in the skin and soft tissue of the chest and shoulder, which are adjacent to the face and neck. Therefore, the branches of the transverse cervical artery are important donor vessels in the reconstruction of the face and neck. However, in recent years, the nomenclature of the flap near the clavicle supplied by the cutaneous branches of the transverse cervical artery has been controversial. Based upon our previous anatomical and clinical research, and after comparison with other nomenclatures, this article proposes a nomenclature based on the anatomy of the region. We believe that the flap near the clavicle supplied by the cutaneous branch of the transverse cervical artery should be referred to as the transverse cervical artery cervical cutaneous branch (TCACCB) flap.

## Anatomical studies

The transverse cervical artery appears stable in anatomy, with a length ranging from 4.0 to 7.0 cm, and a mean diameter of 2.65 mm (1). The transverse cervical vein was present in 61 of 72 cadaveric specimens, with a length ranging from 4.0 to 7.0 cm, and a mean diameter of 2.90 mm (1). As early as the 1940 s, an anatomical atlas suggested that the superficial branch of the transverse cervical artery might supply blood to the skin near the clavicle (3). In 1979, Lamberty confirmed through anatomical and clinical research that the skin in the supraclavicular region was supplied by a stable cutaneous branch of the transverse cervical artery and that this skin could be made into an axial flap (3). He first referred to this flap as the “supra-clavicular axial patterned flap” (3). However, early anatomical studies only found the acromion branch among the cutaneous branches of the cervical segment of the transverse cervical artery, and research on the upper thoracic branch is limited.

Since 1988, we have performed detailed autopsies of the flap near the clavicle supplied by the cutaneous branch of the transverse cervical artery, and found that this branch at the cervical segment supplied blood to the skin in the anterior chest and supraclavicular regions (2). The transverse cervical artery travels outward to the deep side of sternocleidomastoid muscle and scapulohyoid muscle. The vessel then extends deep into the trapezius muscle and enters the dorsal segment of the back. The supraclavicular artery perforates the intersection of sternocleidomastoid muscle and scapulohyoid muscle. It then extends through the fat of the posterior cervical triangle of the neck and enters the subcutaneous layer of the supraclavicular region. Finally, it separates outward and downward into two branches. The first branch, the deltoid branch, extends into the acromial region. The thoracic branch extends into the anterior infraclavicular and thoracic regions (Schematic diagram of TCACCB flap was showed in Supplementary Figure S1) (4). In addition, according to the definition of perforator flap, we believe that the thoracic cutaneous branch of transverse cervical artery can be considered as a perforator vessel. The results of Chin et al. are similar to those of our research group. They found that the cutaneous branch of the transverse cervical artery emanates from the posterior cervical triangle and not only sends out perforating branches through the trapezius muscle to the skin above but also to the skin of the anterior chest (5).

## Nomenclature

Consequent to more research on the flap near the clavicle supplied by the cutaneous branch of the transverse cervical artery in recent years, this flap has been increasingly used; however, there is still no consensus regarding the nomenclature used to refer to this flap, among different articles, as summarized in Table 1.

**Table 1:** Nomenclature for the flap near the clavicle supplied by the cutaneous branch of the transverse cervical artery.

Nomenclature	Donor vessels*	Region of flaps	Main contributor (Year)	References
Transverse cervical artery cervical cutaneous branch (TCACCB) flap	Acromial and thoracic branch	Region near clavicle and anterior chest	Ma (1993)	(2, 6)
Supraclavicular flap**	Acromial branch	Supraclavicular region	Pallua (1997)	(7)
Flap pedicled by the thoracic branch of supraclavicular artery	Thoracic branch	Upper chest region	Xie (2012)	(8)
Anterior supraclavicular artery perforator flap	Thoracic branch	Upper chest region	Pallua (2013)	(9)

*According to the classification of the branch direction of the cutaneous branch of the transverse cervical artery.

**Other names with the same meaning include: supraclavicular island flap, supraclavicular fascial island flap, supraclavicular artery flap, and supraclavicular artery island flap.

Nutrient vessels to transverse cervical artery cervical cutaneous branch (TCACCB) flap are “the cutaneous branch of transverse cervical artery”. The TCACCB flap we propose includes the supraclavicular flap. The extent of the supraclavicular flap in the past was limited to the supraclavicular region (10). The TCACCB flap covers the region near the clavicle and anterior chest; therefore, the scope of the TCACCB flap is larger. The name of the TCACCB flap was first proposed by us, and compared to other names in the literature, not only was it proposed earlier, but it was also more consistent with the anatomic distribution of the blood vessels; therefore, it is a more rational name. Other reasons for proposing this nomenclature are as follows:

• The blood vessels are homologous. The supraclavicular and TCACCB flaps originate from the transverse cervical artery. Mizerny et al. found cutaneous branches of the transverse cervical artery supplying blood to the region near the clavicle by perfusing methylene blue into the transverse cervical artery (11).
• Due to the limitations of the study, the anterior thoracic branch was not dissected, which may be related to issues in the imaging method (12).
• Our study has proven that there are branches from the cervical segment of the transverse cervical artery to the shoulder and anterior chest, and anastomoses between the branches of the transverse cervical artery and the anterior perforating branch of the internal thoracic artery and the thoracoacromial artery have also been found (4).
• It has been proven in our clinical applications that the practical usable range of TCACCB flaps includes the region near the clavicle and anterior chest (13).

## Discussion

The TCACCB flap can be used for the following clinical applications: (1) Neck scar or defect: The TCACCB flap, which is similar to the skin of the neck, can provide a large flap with a matching color and texture, yielding a good clinical effect after repairing neck scars or defects (14). If pre-expansion is performed, the flap area can be significantly increased, the flap becomes thinner, and the donor area does not require skin grafting for repair (15). (2) Chin defect: Choi et al. used the propeller TCACCB flap to repair a defect after resection of a mass in the chin (16). (3) Facial scar: Since the pedicle of the TCACCB flap is located closer to the face, the flap could be designed as an island flap to repair facial scars (17). (**4**) Oropharyngeal defects: Wang et al. used a TCACCB flap to repair oropharyngeal defects after resection of head and neck squamous cell carcinoma and obtained satisfactory repair results (18). Yang et al. also used this flap to repair a tracheal-laryngeal-hypopharynx defect after thyroidectomy with satisfactory results (19). (**5**) Since there is extensive anastomosis between the cutaneous branch of the cervical segment of the transverse cervical artery and the cutaneous branch of the thoracoacromial artery, as well as the second and third perforating branches of the internal thoracic artery, a trans-regional blood supply flap can be designed across regions with a single blood supply artery (20, 21).

For better clinical results, the TCACCB flap was combined with tissue expansion, such that the donor area was increased, and the flap was made thinner and more compatible with the recipient area (22–24). Xu et al. placed expanders of 300 or 350 ml in the anterior thoracic region and over-injected them 2–3 times. After expansion, a 20–25 × 7 cm–9 cm expanded TCACCB flap was obtained for the repair of facial scars, and the donor site could be directly sutured (25). Hou et al. expanded the usable area of the expanded TCACCB flap, using the expanded thoracic flap to repair the donor site, and obtained satisfactory curative effects in the repair of large-area facial and neck scars (26).

The advantages of the TCACCB flap include the following: (**1**) Rich blood supply: Because of the axial blood supply of this flap, the overall blood supply is good, and it can survive well even if there are superficial scars in the donor site. The flap is similar to the face and neck in color, texture, and thickness and has a good postoperative appearance, which is accepted by patients. (**2**) Convenient rotation: Since an island flap is formed, it could be rotated 90–180° without leaving a dog ear. (**3**) Ease of operation: Because of the clear structure of the soft tissues in the neck and chest, the flap can be separated under the deep fascia, making it easy to operate. **(4)** Good sensation: The flap contains the supraclavicular nerve; therefore, the flap retains sensation even after surgery (27).

Although we previously studied the anatomy and clinical application of the TCACCB flap in our department, the name of this flap has always been controversial (28). The absence of uniform names for this flap not only causes confusion in the recognition of the TCACCB flap by plastic surgeons, but also hinders wider applications of this flap. Therefore, we believe that the flap near the clavicle supplied by the cutaneous branch of the transverse cervical artery should be universally referred to as the TCACCB flap. This nomenclature will help the academic community reach a consensus on the naming of this flap, which will greatly promote its clinical application.
