# Supplementary figures and images for: The transverse cervical artery cervical cutaneous branch flap: An anatomy-based nomenclature
Source: Front Surg. 2023 Jan 6;9:1029065. doi: 10.3389/fsurg.2022.1029065 (PMC9856184; doi:10.3389/fsurg.2022.1029065)

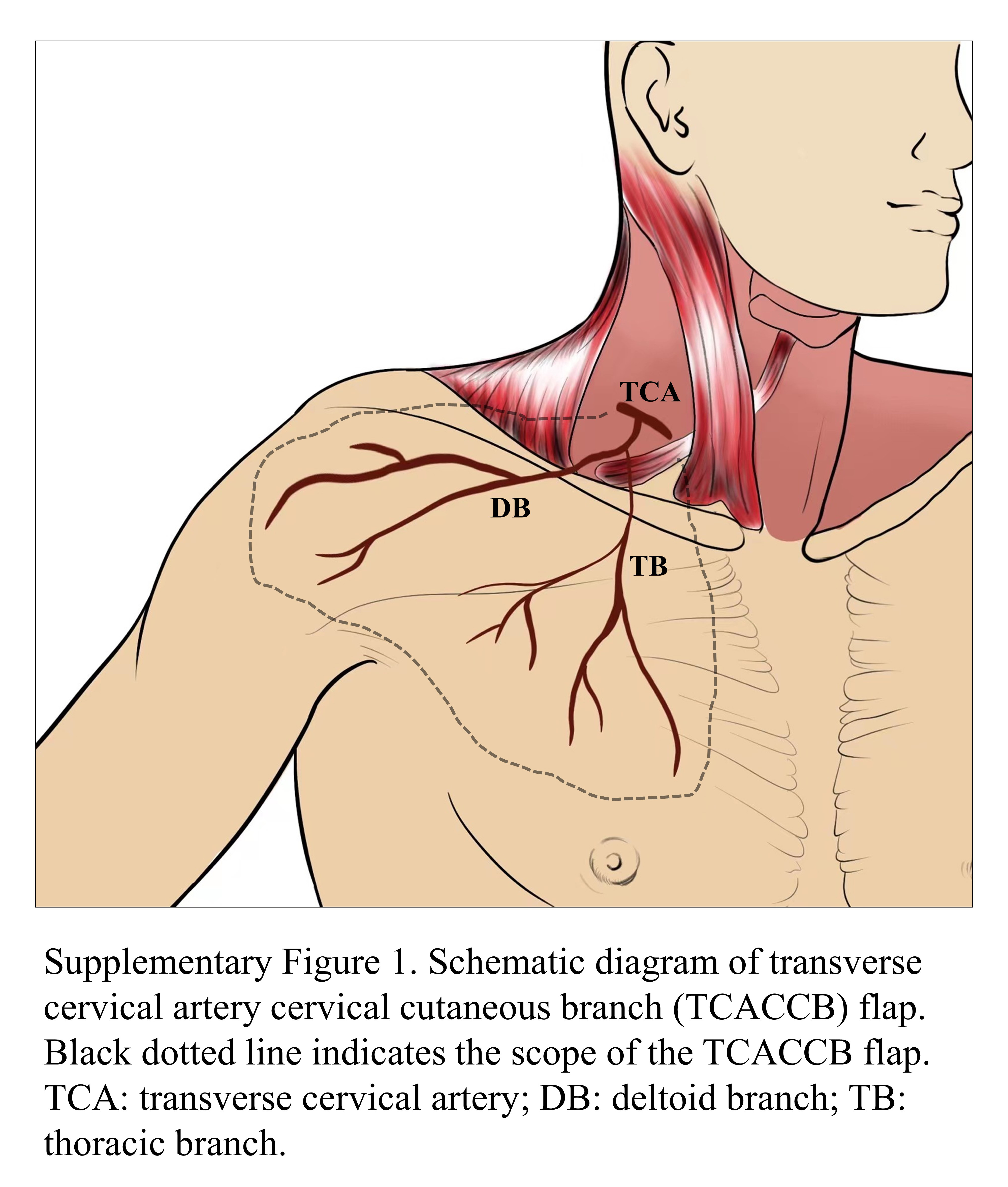

Supplement: Supplementary file 1 [file Image1.jpeg]
